# The Treg/Th17 Axis: A Dynamic Balance Regulated by the Gut Microbiome

**DOI:** 10.3389/fimmu.2015.00639

**Published:** 2015-12-17

**Authors:** Sara Omenetti, Theresa T. Pizarro

**Affiliations:** ^1^Department of Pathology, Case Western Reserve University School of Medicine, Cleveland, OH, USA

**Keywords:** T-regulatory cells, T-helper 17 cells, intestinal homeostasis, gut microbiome, inflammatory bowel disease

## Abstract

T-helper 17 (Th17) and T-regulatory (Treg) cells are frequently found at barrier surfaces, particularly within the intestinal mucosa, where they function to protect the host from pathogenic microorganisms and to restrain excessive effector T-cell responses, respectively. Despite their differing functional properties, Th17 cells and Tregs share similar developmental requirements. In fact, the fate of antigen-naïve T-cells to either Th17 or Treg lineages is finely regulated by key mediators, including TGFβ, IL-6, and *all-trans* retinoic acid. Importantly, the intestinal microbiome also provides immunostimulatory signals, which can activate innate and downstream adaptive immune responses. Specific components of the gut microbiome have been implicated in the production of proinflammatory cytokines by innate immune cells, such as IL-6, IL-23, IL-1β, and the subsequent generation and expansion of Th17 cells. Similarly, commensal bacteria and their metabolites can also promote the generation of intestinal Tregs that can actively induce mucosal tolerance. As such, dysbiosis of the gut microbiome may not solely represent a consequence of gut inflammation, but rather shape the Treg/Th17 commitment and influence susceptibility to inflammatory bowel disease. In this review, we discuss Treg and Th17 cell plasticity, its dynamic regulation by the microbiome, and highlight its impact on intestinal homeostasis and disease.

## Introduction

The gastrointestinal tract represents the largest surface area of the human body that comes into direct contact with the external environment. Consequently, the gut mucosa is exposed to a massive amount and diverse range of foreign antigens. Host detection of pathogenic microbes by antigen-presenting cells (APCs) results in cytokine production, as well as recruitment and differentiation of T-helper (Th) cells. The nature of the offending organisms is crucial for the differentiation into Th cells, and once contained, effector responses are counterbalanced by Tregs that limit collateral damage. In peripheral organs, such as the gut, the balance between Treg/effector cells is normally achieved by *in situ* induction of these cells from naïve T-cells, recruitment of differentiated Treg/effector cells into the tissue, and reprogramming of already differentiated Treg/effector cells towards other lineages in peripheral tissues (1, 2).

## Treg/Th17 Axis in Health and Disease

### Treg and Th17 Cells: Similarities Beyond Functional Opposites

Th17 cells have only recently been identified as a unique CD4^+^ T-helper subset, characterized by IL-17 production that promotes tissue inflammation (3, 4). Understanding their function during homeostatic and inflammatory conditions is continuously evolving; however, it is increasingly clear that Th17 cells are critical in protecting mucosal surfaces against microbial pathogens, including bacteria, fungi, and viruses (5, 6), particularly in the lamina propria (LP) of the small intestine (SI), where they are abundantly present (7). Notoriety of Th17 cells initially emerged with the report that IL-17-producing T-cells, driven by IL-23, were major contributors to autoimmune inflammation (8). Indeed, the initial discovery that IL-23, rather than IL-12, was required to develop disease in experimental models of inflammation (9, 10) led to the reevaluation of T-cells that drive IL-23-dependent inflammation.

Over the last two decades, Tregs have been identified as dedicated suppressors of diverse immune responses and inflammation, and central keepers of peripheral tolerance. Tregs are generated in both the thymus (natural Tregs and nTregs) and the periphery (iTregs). While iTregs resemble nTregs in phenotype and function, there are also differences, most prominently regarding their epigenetic and transcriptional status, as well as their inherent stability (11–13). Indeed, when naïve CD4^+^ T-cells recognize antigen presented as self, in the absence of any inflammatory stimuli, tolerance is induced and these cells, at least partially, differentiate into Tregs. Accordingly, organs exposed to a wide repertoire of foreign antigens, such as the gut, may be dominated by Tregs arising from peripheral conversion, rather than thymic-cell differentiation. The peripheral antigenic landscape may also affect selective expansion of Treg T-cell receptor (TCR) clonotype (14) that is presumably dependent on a peripheral antigenic niche (15). According to this scenario, iTregs represent an essential, non-redundant regulatory subset that supplements nTregs, in part by expanding TCR diversity (16).

Although Tregs and Th17 cells fundamentally differ in function, they also display many common features. Both populations are abundantly found in the periphery, particularly in the intestine (7, 17), and are composed of heterogeneous subpopulations that are able to change effector or suppressor capabilities under different conditions (2). Moreover, shared mechanisms and key mediators (e.g., lineage-specific transcription factors, cytokines) regulate Th17 cells and Tregs, similar to other T-helper subsets. The pleiotropic cytokine, TGFβ, for example, is essential for differentiation of both cell types. TGFβ is non-redundantly required for the generation of Tregs (18) but dispensable for the development of Th17 cells (19). IL-1β can substitute TGFβ in IL-6-mediated generation of Th17 cells (20). Thus, in the absence of proinflammatory signals, such as IL-6 produced by microbial-activated dendritic cells (DCs) or IL-21 produced by IL-6-stimulated T-cells (21–23), priming of naïve CD4^+^ T-cells by antigen in an environment rich in TGFβ promotes the development of iTregs (24, 25). Conversely, activation in an environment wherein both TGFβ and IL-6 are available promotes Th17 development, at least at mucosal sites (26).

Nonetheless, it is perplexing how the same cytokine can regulate differentiation of T-cells with opposing functions. The answer likely lies in TGFβ’s concentration-dependent function. At low concentrations, TGFβ synergizes with IL-6 and IL-21 to promote IL-23 receptor (IL-23R) expression, favoring Th17 differentiation (21–23), whereas at high concentrations, TGFβ represses IL-23R and favors Foxp3^+^ Tregs, which in turn inhibits RORγt function (27). Conversely, IL-21 and IL-23 can relieve Foxp3-mediated inhibition of RORγt, thereby promoting Th17 differentiation (27). Therefore, the decision of antigen-stimulated cells to differentiate into either Th17 or Tregs depends upon the cytokine-regulated balance of the two master regulators of these cells, RORγt and Foxp3, respectively. Several other mediators can also influence the balance between Th17 and Tregs. RA, a metabolite of vitamin A, preferentially induces Tregs over Th17 cells by enhancing TGFβ signaling while blocking IL-6R expression (28). Moreover, aryl hydrocarbon receptor (AhR), highly expressed on Th17 and Tregs, can promote the induction of both cell types by integrating environmental stimuli (29, 30). Environmental stimuli affecting gastrointestinal immunity via AhR can consist of both dietary- and bacteria-produced ligands, which can interact directly with AhR (31, 32). Interestingly, the loss of bacteria-producing AhR ligands may influence gut immunity and increase the risk of colitis (33).

### Treg/Th17 Plasticity

Several studies have established that differentiation of Foxp3^+^ Tregs is not static and that Tregs can differentiate into Th17 cells. This phenomenon was first reported in mice, wherein IL-6 was shown to convert Foxp3^+^ cells to Th17 cells in the absence of TGFβ (34–36), which was confirmed in humans (37, 38). In contrast to “Th1-like” Tregs, IL-17-secreting Tregs are suppressive *in vitro* but lose this capacity upon stimulation with IL-1β and IL-6 (38). Accordingly, among RORγt^+^Tαβ cells derived from different murine tissues, the presence of Foxp3^+^cells that function as Tregs has been reported that coexist with IL-17-producing RORγt^+^Tαβ cells (39). In this study, the ratio of Foxp3^+^ to IL-17-producing RORγt^+^Tαβ cells is skewed in favor of IL-10 production by Foxp3 and CCL20 and in favor of IL-17 by IL-6 and IL-23. It is unclear why only some IL-17^+^ cells express Foxp3, and how this is biological relevant. It is possible that Foxp3 activation occurs during Th17 programming, or alternatively, that Foxp3 expression may signify a distinct differentiation pathway. A recent report showed that under arthritic conditions, CD25^lo^Foxp3^+^CD4^+^ T-cells lose Foxp3 expression and undergo IL-6-dependent transdifferentiation into Th17 cells, which accumulate in inflamed joints. Once adoptively transferred into mice, these cells are able to accelerate the onset, and increase severity, of arthritis and associate with loss of Foxp3 expression in the majority of transferred T-cells (40). Interestingly, IL-17-producing Foxp3^+^CD4^+^ lymphocytes are also observed in inflammatory bowel disease (IBD) patients (41). These cells share phenotypic characteristics with both Th17 and Tregs and show potent *in vitro* suppressor activity (42) and increased sensitivity to Th17-generating cytokines in IBD patients versus controls (41). Although Tregs are not sufficient at controlling inflammation in IBD, it is unclear whether or not they retain their suppressive function. Increasing, albeit confounding, evidence points to the different cell origins responsible for this discrepancy (43, 44), adding further complexity to the biological relevance of the functional and phenotypic overlap between Treg and Th17 cells observed in IBD (41, 42).

Whether Th17 cells represent a terminally differentiated lineage or a metastable state is still an area of debate. Multiple studies have identified a Th17 subset that coproduces IFNγ, such as in the inflamed intestine, where they display developmental plasticity (45, 46). Generally, Th17 cells can retain an IL-17A^+^ phenotype, or lose IL-17 and acquire expression of IFNγ, in a process driven by IL-12 and IL-23 via a STAT4- and T-bet-dependent manner (47), thus giving rise to Th1-like cells. The latter Th17 subset does not possess colitogenic potential, whereas the former, derived from a Th17 precursor, can mediate experimental colitis via STAT-4 and T-bet, but not through IL-2 or IFNγ receptors (48). One reason that Th17 cells display considerable developmental plasticity may be that RORγt does not participate in stabilizing positive feedback toward transcription factor activation, thus rendering its expression sensitive to environmental signals (49).

**Figure 1:**
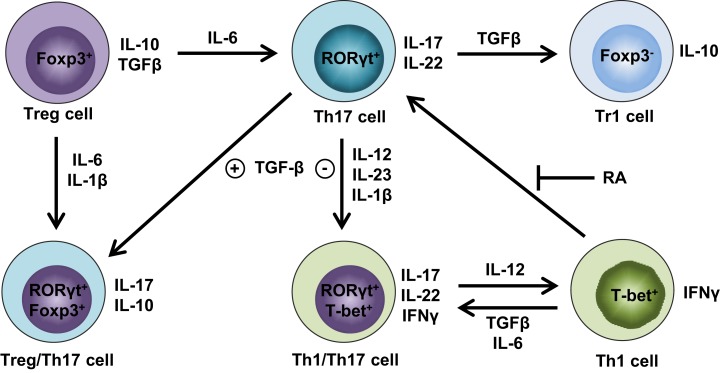
**Cytokine milieu orchestrates Treg and Th17 cell plasticity**. Th17 cells lose stability in the absence of TGFβ and presence of IL-12, IL-23, and IL-1β, favoring IFNγ expression and differentiation into Th1/Th17 cells that produce both Th1 (e.g., IFNγ) and Th17 (e.g., IL-17, IL-22) cytokines. Further augmentation of IL-12 can fully convert Th1/Th17 cells into Th1 cells, whereas this process can be reverted by either TGFβ and IL-6 or in the absence of retinoic acid (RA) in favor of Th1/Th17 or Th17 cells, respectively. Alternatively, the abundance of TGFβ in the absence of IL-6 drives Th17 cells toward regulatory phenotypes, such as either RORγt^+^Foxp3^+^ Treg/Th17 cells or Foxp3^−^ Tr1 cells. If proinflammatory cytokines are present, including either IL-6 or IL-1β and IL-6, Foxp3^+^ Tregs have the ability to transdifferentiate into either Th17 or Treg/Th17 cells, respectively.

Until recently, the conversion between Treg and Th17 was thought to be a one-way street, wherein Tregs can unidirectionally convert into Th17 cells (34). Although coexpression of Th17 and Treg signature genes has been reported in the same cells (38, 39), it is unclear whether Th17 cells can undergo a global reprogramming that drives conversion to another Th-type or that they simply display phenotypic plasticity. Gagliani et al. made the seminal discovery that under homeostatic conditions, intestinal Th17 cells can lose IL-17 expression and a fraction of these “exTh17” cells acquire regulatory features resembling CD4^+^Foxp3^−^ Type 1 Tregs (Tr1) (50). This conversion is determined by reprogramming of the Th17-relevant transcriptional profile, referred to as “transdifferentiation” (51, 52). The functional reprogramming experienced by exTh17 into Tr1 is irreversible; indeed, these cells display anti-inflammatory properties by preventing Th17-mediated colitis (50). While Th17 cells generated with TGFβ1/IL-6/IL-23 are able to promote colitis, exTh17 Tr1 cells generated under the same conditions fail to induce disease. In fact, although TGFβ1 is important for exTh17 Tr1 cell development, Th17 cells remain colitogenic, despite the presence of TGFβ1, as long as they do not convert into Tr1 cells (50). While the main cytokines orchestrating Treg/Th17 plasticity have been identified (Figure 1), the fine balance of environmental stimuli required for directing T-helper cells toward one lineage versus another is still known.

## Regulation of the Treg/Th17 Axis by the Gut Microbiome

### Metabolic Control of Th17 and Tregs

Environmental signals and microbiome sensors can profoundly affect T-cell differentiation and response to immune stimuli. Generally, activated effector T-cells are anabolic, primarily employing glucose as their carbon source and utilizing glycolysis for fast access to adenosine triphosphate (ATP). Memory and resting T-cells are instead catabolic with the ability to metabolize fatty and amino acids, in addition to glucose, and depend on oxidative phosphorylation to generate ATP (53). Two key mediators of the glycolytic and lipogenic pathways in T-cells are mammalian target of rapamycin (mTOR) and adenosine monophosphate-activated kinase (AMPK), which promote *de novo* fatty acid synthesis and fatty acid oxidation, consequently inducing either energy production or storage, respectively (54). Both mTOR and AMPK act as crucial cellular energy sensors and are regulated by the availability of nutrients (55, 56). Th17 cells depend on acetyl-CoA carboxylase 1-mediated *de novo* fatty acid synthesis and the underlying glycolytic-lipogenic metabolic pathway for their development, whereas Tregs rely on oxidative phosphorylation and consume their required fatty acids exogenously (57). Upregulation of the glycolic pathway in Th17 cells can also be activated by the transcription factor, hypoxia-inducible factor 1α (HIF1α) (58) that binds to the locus encoding RORγt and enhances its expression while inhibiting Foxp3. Together, this promotes T-cell differentiation toward Th17 and prevents Treg commitment under both normoxic and hypoxic conditions (59). Differently from Foxp3^+^ Tregs, Tr1 metabolism is supported by glycolysis via HIF1α in early metabolic reprogramming and by AhR at later stages, which then promotes HIF1α degradation (60). Both hypoxia and extracellular ATP increased at inflammatory sites (61, 62), triggered AhR inactivation, and inhibited Tr1 differentiation (60). Therefore, metabolic factors present in the microenvironment have immune-modifying potential, which can skew the balance between inflammation and immune tolerance by biasing the decision of T-cell fate toward either Th17 or Treg lineages.

### Regulation of Th17 and Treg by the Commensal Flora

The importance of the gut microbiome in regulating the Treg/Th17 axis became widely appreciated when different groups reported that germ-free (GF) mice demonstrate a decreased frequency of SI Th17 cells and colonic Tregs (63, 64). One of the most widely investigated commensal bacteria in the context of Th17 immunity is segmented filamentous bacteria (SFB), a *Clostridia*-related species (65) that displays features between an obligate and facultative symbiont (66), suggesting that these bacteria obtain nutritional requirements from their host (65). Interest peaked when SFB was reported to specifically induce Th17 cells in the SI (67, 68) and in extraintestinal sites during autoimmune inflammation (69, 70). SFB antigen is presented to CD4^+^ T-cells by DCs in a major histocompatibility complex-dependent manner, which is required for the induction of SFB-specific intestinal Th17 cells (71, 72). SFB colonization of GF mice activates a wide range of antimicrobial defenses, including immunoglobulin (Ig)A secretion and LP production of antimicrobial peptides and proinflammatory cytokines (63, 67, 68). SFB colonization is potentially beneficial since it attenuates bacteria-induced colitis (68), but it can also induce colitis in genetically susceptible mice (73), suggesting that while SFB can normally enhance immune control of infection, its presence can also result in inflammation. The abundance of SFB, together with gut barrier function, is regulated by the IL-23R/IL-22 pathway (74). When the intestinal barrier is disrupted, systemic dissemination of microbial products occurs, which invokes the IL-23 pathway and initiates barrier repair, as well as Th17 responses aimed to neutralize invading commensal microbes (74). Moreover, SFB-induced-IL-23 results in production of IL-22 by type 3 innate lymphoid cells, which is critical for the production of serum amyloid A proteins 1 and 2 by epithelial cells (75). This circuit promotes IL-17 expression in RORγt^+^ T cells, especially in the terminal ileum, which is the site of SFB attachment to the epithelium, the essential condition for Th17 induction by SFB (75, 76). SFB-induced activation may also result in the generation of autoreactive Th17 cells in response to presentation of autoantigen in the setting of a breached intestinal barrier. SFB-mediated induction of Th17 immune responses can also occur indirectly via other cell types. Indeed, Treg-specific MyD88 deficiency is sufficient to impair intestinal IgA responses to SFB and results in the expansion of Th17 cells (77).

Another resident of the human gut microbiome influencing T-cell homeostasis is the symbiont, *Bacteroides fragilis*. *Bacteroides* species are normal constituents of the intestinal microbiome; however, under certain circumstances, these microbes can become pathogens. Polysaccharide A (PSA), the most abundant capsular polysaccharide expressed by *B. fragilis*, mediates conversion of CD4^+^ T-cells into IL-10-producing Foxp3^+^ Tregs via TLR2 and suppresses Th17 responses, thus facilitating colonization of *B. fragilis* (78). Consistently, PSA is able to both prevent and ameliorate experimental colitis (79), suggesting that *B. fragilis* facilitates Treg differentiation in the gut and induces mucosal tolerance. Nevertheless, strains of *B. fragilis* secreting the zinc metalloprotease, *B. fragilis* toxin (BFT), have been implicated in IBD (80, 81) and in IL-17-dependent inflammation-associated colon cancer (82). Indeed, BFT can alter the function of intestinal epithelial tight junctions, resulting in increased permeability and diarrhea (83).

**Figure 2:**
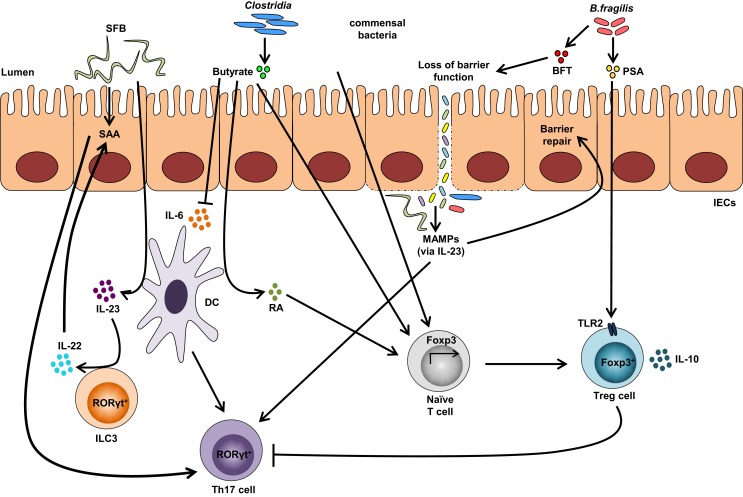
**Impact of the gut microbiota on Treg and Th17 immune responses**. Colonization with segmented filamentous bacteria (SFB) occurs by intimate attachment to the intestinal epithelium and promotes the development of Th17 cells via intestinal epithelial cell (IEC)-derived cytokines, serum amyloid A (SAA), as well as antigen presentation by dendritic cells (DCs). Adhesion of SFB to IEC can potentially generate a circuit, wherein DC-derived IL-23 stimulates IL-22 production by type 3 innate lymphoid cells (ILC3), which in turn induces SAA from IEC and can lead to Th17 cell differentiation. Conversely, colonization of beneficial commensal bacteria induces *de novo* generation of Tregs and downregulates Th17 immune responses. Commensal bacteria, including most *Clostridia* species, produce short-chain fatty acids, i.e., butyrate, which participates in the *de novo* generation of Tregs by suppressing proinflammatory cytokines, by promoting RA production from DCs, and by inducing Foxp3 transcription. Among different *Bacteroides fragilis* strains, those expressing polysaccharide A (PSA) mediate the generation of Tregs via TLR2, while those secreting *B. fragilis* toxin (BFT) alter the function of IEC tight junctions. Upon disruption of barrier function, dissemination of microbial products, recognized by microbe-associated molecular patterns (MAMPs), occurs and activates the IL-23 pathway, resulting in subsequent barrier repair and stimulation of Th17 immune responses.

Other bacterial strains, such as *Clostridia*, are able to induce Tregs within the gut. Most *Clostridia* maintain a commensal relationship with the host, with a few exceptions, including *Clostridia perfringens*, *Clostridia difficile*, and *Clostridia tetani*, which produce toxins and are pathogenic. Colonization of GF mice with a defined mixture of 46 *Clostridium* strains belonging to clusters XIVa and IV induces the differentiation of colonic Helios-negative Tregs in a MyD88-independent manner (64). Additionally, a mixture of 17 strains from *Clostridiales* clusters VI, XIVa, and XVIII isolated from human feces also exhibits Treg-inducing activity (84) and suggests that *Clostridium*-dependent induction of Tregs may contribute to the maintenance of intestinal immune homeostasis. Similarly, colonization of GF mice with altered Schaedler flora, a standardized cocktail of benign intestinal commensal microbiota, results in the *de novo* generation of Tregs and downregulation of Th1 and Th17 immunity (85). A summary of bacterial strains influencing Treg and Th17 intestinal immune responses are depicted in Figure 2.

The precise mechanism(s) underlying colonic Treg induction by the gut microbiome remains unclear, although several reports suggest that commensal, microbe-derived short-chain fatty acids (SCFAs), particularly those from *Clostridiales*, may be involved (86–88). SCFAs, together with organic acids and alcohols, are metabolic end products generated in the lower gastrointestinal tract from fermentative growth of carbohydrates and proteins that cannot be degraded (89). Specifically, locally produced butyrate participates in colonic *de novo* Treg development, whereas oral administration of acetate and propionate contributes to Treg migration into the colon by upregulating G-protein receptor (GPR)15 (90). Indeed, *in vivo* administration of butyrate suppresses proinflammatory cytokines from macrophages and DCs, likely through inhibition of histone deacetylases (87, 91), and ameliorates experimental colitis (88). Butyrate participates in Treg differentiation by facilitating histone H3 acetylation in the promoter and conserved non-coding sequence regions 1 and 3 of the Foxp3-encoding locus (88) or by activating its receptor, GPR109a, that promotes RA production in DCs and leads to induction of Treg differentiation (92). Interestingly, T-cell regulation by SCFAs is dependent on the cytokine milieu and immunological context. Indeed, acetate promotes IL-10-producing T-cells during steady-state conditions and effector Th1 and Th17 cells during active immune responses (93). Other dietary-related factors, such as fat-enriched diets, have been implicated in gut microbial regulation of intestinal immunity. In fact, high-fat-diet-derived microbiota decreases Th17 cell frequency and the ability of intestinal APCs to generate Th17 cells *in vitro*, thus contributing to low-grade inflammation (94).

### Dysbiosis Affecting the Th17/Treg Axis in IBD

Dysbiosis is considered an alteration of the resident commensal microenvironment compared to commensal communities found in healthy individuals and has been reported in many diseases, including IBD (95). Dysbiosis can be classified into three different, non-mutually exclusive, categories: loss of beneficial microbial organisms, expansion of pathobionts, and loss of overall microbial diversity (96). Interestingly, reduced abundance of butyrate-producing bacteria, i.e., *Clostridiales* cluster IV and XIVa, has been found in IBD patient fecal samples (95), supporting the hypothesis that presence of beneficial bacteria inducing Treg differentiation is important to prevent IBD. Although there is no single organism capable of inducing IBD, a few pathogens have been implicated. Two colitogenic proteobacteria, *Proteus mirabilis* and *Klebsiella pneumonia*, have been identified in ulcerative colitis-like T-bet^−/−^Rag2^−/−^ mice that spontaneously develop dysbiosis and colitis (97, 98). However, maternally transmitted endogenous microbes are also required to maximize inflammation in these mice (98), suggesting that microbe interaction may determine whether a pathobiont will display a pathogenic profile. Similarly, IBD patients display an increased number of *Actinobacteria* and *Proteobacteria* (95). Intestinal T-cell homeostasis appears to be dependent not only on the type of bacteria present but also on overall microbial diversity. Indeed, the transfer of over 30 different human *Clostridia* strains into GF mice induced a threefold expansion of Tregs compared to uncolonized controls, whereas transfer of a single strain from the same *Clostridia* collection induced a more modest Treg response (64, 84), suggesting that greater microbial diversity maximizes host immune responses and its reduction may contribute to inflammatory processes, such as in IBD (99).

## Conclusion

Although progress has been made in clarifying the role of the microbiome in Treg and Th17 mucosal immunity, its impact on disease, such as IBD, remains controversial. A better understanding of the mechanisms regulating these processes may aid in the development of therapeutic agents aimed to maintain appropriate Treg/Th17 balance and restore homeostatic function during disease states.

## Author Contributions

SO and TP conceptualized review. SO provided an initial draft of the review, while TP performed the final edits.

## Conflict of Interest Statement

The authors declare that the research was conducted in the absence of any commercial or financial relationships that could be construed as a potential conflict of interest.

## Abbreviations

AhR, aryl hydrocarbon receptor; AMPK, adenosine monophosphate-activated kinase; APC, antigen-presenting cell; ATP, adenosine triphosphate; BFT, *Bacteroides fragilis* toxin; DC, dendritic cell; GF, germ-free; GPR, G-protein receptor; HIF1α, hypoxia-inducible factor 1α; IBD, inflammatory bowel disease; IL-23R, IL-23 receptor; Ig, immunoglobulin; iTreg, induced Treg; LP, lamina propria; mTOR, mammalian target of rapamycin; nTreg, natural Treg; PSA, polysaccharide A; RA, retinoic acid; SCFA, short-chain fatty acid; SFB, segmented filamentous bacteria; SI, small intestine; TCR, T-cell receptor; Th, T-helper; Treg, T-regulatory cell; Tr1, T-regulatory type I cell.
